# SmartFPS: Neural network based wireless-inertial fusion positioning system

**DOI:** 10.3389/fnbot.2023.1121623

**Published:** 2023-02-10

**Authors:** Luchi Hua, Yuan Zhuang, Jun Yang

**Affiliations:** ^1^National Application Specific Integrated Circuit Center, Southeast University, Nanjing, China; ^2^State Key Laboratory of Information Engineering in Surveying, Mapping, and Remote Sensing, Wuhan University, Wuhan, China; ^3^Hubei Luojia Laboratory, Wuhan, China; ^4^Wuhan University Shenzhen Research Institute, Shenzhen, China

**Keywords:** indoor positioning, wireless positioning, Kalman filter, deep learning, transfer learning

## Abstract

Current wireless-inertial fusion positioning systems widely adopt empirical propagation models of wireless signals and filtering algorithms such as the Kalman filter or the particle filter. However, empirical models of system and noise usually have lower accuracy in a practical positioning scenario. The biases of predetermined parameters would enlarge the positioning error through layers of systems. Instead of dealing with empirical models, this paper proposes a fusion positioning system based on an end-to-end neural network, along with a transfer learning strategy for improving the performance of neural network models for samples with different distributions. Verified by Bluetooth-inertial positioning in a whole floor scenario, the mean positioning error of the fusion network was 0.506 m. The proposed transfer learning method improved the accuracy of the step length and rotation angle of different pedestrians by 53.3%, the Bluetooth positioning accuracy of various devices by 33.4%, and the average positioning error of the fusion system by 31.6%. The results showed that our proposed methods outperformed filter-based methods in challenging indoor environments.

## 1. Introduction

Location navigation service is one of the indispensable technologies for the modern society and scientific development. However, the current Global Navigation Satellite System (GNSS) is usually unable to locate effectively indoors due to irregular attenuation of GNSS signals caused by the occlusion of clouds, building walls, and ceilings. During the last decades, many efforts have been made to study how to leverage any indoor wireless signals to provide indoor navigation services, and indoor positioning technology was born. At present, mainstream indoor positioning technologies include Wi-Fi (Zhuang et al., 2016), Bluetooth (Paterna et al., 2017), Ultra-wide Band (UWB) (Mahfouz et al., 2008), Lidar (Li et al., 2020), computer vision (Hwang and Song, 2011), inertial navigation (Kang and Han, 2015; Wu et al., 2019; Hou and Bergmann, 2021), visible light (Zhuang et al., 2018, 2019; Hua et al., 2021) and so on. Each positioning technique has its advantages as well as its limitations. For example, inertial navigation positioning is prone to accumulative errors due to system noise and drift (Abdulrahim et al., 2010). Modeling signal propagation is challenging for Wi-Fi and Bluetooth since they are easily affected by occlusions, and traditional positioning algorithms, such as trilateration positioning (Yang et al., 2020), usually have low positioning accuracy. The current UWB devices are too expensive to be widely promoted (Alarifi et al., 2016); Lidar is also costly and has specific requirements for wall reflection coefficient (Roche et al., 2022), so it would fail under poor lighting conditions. Generally, low-cost, high-precision and high-stable positioning performance cannot be obtained based on a single positioning system. Therefore, most practical positioning solutions leverage multiple sensor data from the user's mobile devices, such as the gyroscope and accelerometer of the inertial unit, Wi-Fi, and Bluetooth modules in the smartphone. Many fusion positioning systems have been proposed in recent years, such as the fusion of Wi-Fi, Bluetooth, Lidar, and inertial sensor (Chen et al., 2014; You et al., 2020; Yang et al., 2022).

Current wireless-inertial fusion positioning systems widely adopt empirical propagation models of wireless signals and filtering algorithms such as the Kalman filter (KF) or the particle filter (PF). However, in many practical positioning scenarios, the complexity of the fusion system or a single system is usually very high. For example, for pedestrian navigation, it is quite challenging to model a step-heading estimation system (SHS) or the distribution characteristics of wireless signals in the environment. Besides, the increase in the system complexity will enlarge the influence on the parameter error to the final state estimation. Although many studies apply machine learning techniques to deal with the complexity of a single positioning system, fusion is still based on filtering algorithms. However, it is rather challenging to determine the noise parameters in the filtering methods, especially the noise of the wireless positioning, which is irregularly distributed in the whole area. If wireless positioning adopts the fingerprinting method, the noise should be analyzed area by area. Furthermore, by assigning specific values to the covariance matrixes of process and observation, the accuracy of the noise parameter is compromised. Considering those factors, deep learning is a more straightforward way to accurately model the noise and build the whole model at the same time. In this study, we propose a fusion localization system based on an end-to-end neural network and a transfer learning strategy for improving the performance of the neural network model for samples with different distributions. Furthermore, we verified our methods in a Bluetooth-inertial fusion positioning scenario considering the low cost, quick deployment of the commercial Bluetooth beacons, and the convenience of leveraging built-in smartphone sensors. Our contributions are summarized as follows:

1) An end-to-end trainable neural network-based inertial-wireless fusion positioning system, namely SmartFPS, is first proposed. Besides, a training procedure based on multi-task learning is also proposed. Large field experiments were performed, and results showed that our method could outperform filter-based fusion systems by 36.5% when optimal parameters were set for the particle filter.
2) A transfer learning strategy for SmartFPS is proposed based on the generative adversarial network (GAN) to deal with device heterogeneity and other factors in practical positioning scenarios. Simultaneously training the GAN-based network with labeled data in the source domain and the unlabeled data in the target domain improved the positioning accuracy by over 50%, compared with training SmartFPS with the source domain dataset.

## 2. Related works

In wireless-inertial fusion localization systems, the processing and modeling of inertial sensors, wireless signals, and their fusion method must be carefully considered. In recent decades, inertial navigation and indoor wireless positioning have been studied individually, from empirical modeling to machine learning. Fusion methods, especially filtering methods, have also improved to cope with complex processes and non-Gaussian noise. This section will discuss the development of these three components in fusion positioning separately to show the advantages of replacing all empirical models with deep learning.

### 2.1. Pedestrian inertial navigation

Pedestrian inertial navigation mainly includes the strap-down inertial navigation system (SINS) (Bortz, 1971) and the step-heading estimation system (Jirawimut et al., 2003). However, those methods rely on accurate sensor adjustment or estimation of step length and direction, which are often corrupted by noise and drift in inertial systems.

As for SINS, even if there is only a small error in the angle estimation, the final position result will be affected exponentially through multi-layer transfer. The effect of this error is especially severe for low-cost MEMS sensors in smartphones. Usually, the extended Kalman filter (EKF) can be used to lower this error.

One measure to deal with the drift problem is to close the integrating loop periodically by imposing external constraints on the system. The most widely used constraint method is the zero velocity update (ZUPT) (Foxlin, 2005). ZUPT is based on the sensor being at rest and can be applied during the stance phase, provided the sensor is attached to the foot. ZUPT is easily incorporated into the INS structure by representing ZUPT as a pseudo-measurement of zero velocity. By applying ZUPT, the open loop integration only occurs during the swing phase of the foot. For such short durations, the accumulation of drift is limited, so longer tracking durations are feasible. However, for reliable output, ZUPT must only be applied when the foot is completely stationary. Problems can arise when the sensor is mounted higher than the sole. The peeling motion associated with the transition from standing to swinging means that the heel rises soon after the foot lands down, so the sensor in the midfoot will begin to experience acceleration as the foot lifts. These small accelerations occur before the strict end of the stance phase, so it is necessary to account for these errors by applying a non-zero covariance next to the ZUPT pseudo-measurement.

To simplify the modeling of inertial navigation, a deep learning-based method, IONet (Chen et al., 2018), was proposed. Unlike the traditional SHS system, the step length and direction are estimated by training the neural network with sequential inertial data. Since the neural network has an excellent capability of non-linear system modeling, the accuracy of step length and direction estimation is significantly improved. IONet is formed by a two-layer long short-term memory neural network (LSTM), a specific neural network dealing with time series data. The input of the system is the accelerometer and gyroscope time sequence under a fixed time window, and the output is the step length and rotation angle. On the premise that the initial position and direction of the pedestrian are known, the system could estimate the position and direction through each step's estimated step length and rotation angle. Experiment results showed that the neural network-based method significantly improved positioning accuracy compared with the PDR method. The method based on the neural network not only enhances positioning accuracy but also simplifies various complex processes such as data processing and noise analysis.

Inspired by IONet, a new system, namely RoNIN, was proposed to improve the positioning performance of deep learning-based inertial navigation (Herath et al., 2020). This study investigated three network structures: the ResNet network, the LSTM network, and the temporal convolutional network (TCN). Another difference from IONet is that the output of the system proposed in this study is a velocity vector. Acquiring ground truth labels in large environments is challenging, and the study used the Tango system by attaching an Android phone to the front of the body. Another Android phone was held in one hand to collect inertial data. The pre-processing of inertial data was explained in detail in this study. A spatiotemporal calibration process was introduced to calibrate inertial data and ground truth from two devices. Verified by a massive dataset in a large environment, the results showed that the positioning accuracy of the proposed system was much higher than IONet.

### 2.2. Indoor positioning based on wireless signals

Wireless signal-based positioning systems generally build positioning models based on features such as received signal strength (RSS), angle of arrival (AOA), and time of flight (TOF). However, due to the advantage of simple implementation, most wireless-based positioning solutions adopt the RSS feature. RSS-based wireless positioning algorithms generally have three types: proximity (Yu et al., 2020), trilateration, and fingerprinting.

The Proximity method is the simplest positioning method that determines whether the receiving device is close to a wireless beacon according to the RSS value and directly uses the position of the beacon as the position of the receiving device. Therefore, the positioning accuracy of the proximity method is usually low. However, the proximity method has the advantage of high robustness. Although the RSS of the wireless signal will fluctuate, the RSS value after a smoothing process is within a fixed range when the device is very close to the wireless beacon.

The RSS-based trilateration estimates a device's location based on the RSS values of three or more wireless beacons. Due to the fluctuation of RSS, the circles of three distances estimated by the RSS values do not intersect at the same point but overlap in an area. Usually, non-linear optimization methods can solve this position, such as least squares. For trilateration positioning, the positioning accuracy depends on at least three line-of-sight (LOS) wireless signals. In practical positioning scenarios, due to the consideration of deployment costs, the distance between wireless beacons is large, and inevitably, there will be environmental interference or occlusion. Therefore, it is challenging to collect three LOS signals continuously. Even though three LOS signals exist, the signal-to-noise ratio (SNR) is low in specific locations due to high noise and weak signal. As a result, trilateration positioning often has low robustness.

Fingerprint positioning technology is widely used to solve the problem of wireless positioning under a low SNR. Fingerprint positioning technology mainly collects RSS values from different wireless beacons at various locations in the scene in the offline stage, and the combination of these measurement values becomes the fingerprint information of the location. After deploying the system, the RSS combination of online measurements (obtained in real time) is compared with offline measurements to estimate user location. Many machine learning methods, such as K-nearest neighbors (KNN) and support vector machine (SVM), and deep learning methods, such as multi-layer perceptron (MLP), can be used to solve fingerprint matching problems. Fingerprint localization algorithms usually need to survey the environment to obtain fingerprints or characteristics of the environment, so it is also called scene analysis localization technology.

### 2.3. Fusion algorithms based on filtering methods

The filtering algorithm is one of the most widely used fusion algorithms in mainstream indoor fusion positioning systems. At present, the filtering algorithms applied in the positioning fusion algorithm mainly include (1) the discrete Bayesian filter (Fox et al., 2003); (2) the Kalman filter; (3) the particle filter (Chen et al., 2022). The Kalman filter also includes the extended Kalman filter (Ozbek et al., 2010), the unscented Kalman filter (UKF) (You et al., 2020), and so on. All of the filters have their advantages and drawbacks.

The Kalman filter is theoretically the best estimate for unimodal linear systems with Gaussian noise but not for non-Gaussian non-linear systems. To address this limitation, various improved Kalman filter algorithms, such as the extended Kalman filter and the unscented Kalman filter, were proposed in subsequent research.

Since the extended Kalman filter is a first-order estimation for non-linear systems, it cannot achieve good performance for strongly non-linear systems. Therefore, second-order and third-order extended Kalman filters are proposed. In addition, the extended Kalman filter cannot guarantee the convergence of the algorithm. The algorithm will diverge if the initial state quantity error is significant or the process model is incorrect.

UKF can handle non-linear, continuous, multivariate problems. The sigma points specifically proposed in UKF can also estimate a certain degree of non-Gaussian noise but cannot accurately estimate complex non-Gaussian distribution.

The particle filter can handle non-linearity and non-Gaussian noise. However, at different degrees of non-linearity and non-Gaussian noise, the number of particles, the particle generation strategy, and the resampling strategy can significantly affect the accuracy. Besides, more particles will increase the computational cost and lower the calculation speed. In addition, the performance of particle filters in dealing with high-dimensional systems is unstable because high-dimensional systems can easily lead to excessive differences in the weight distribution of particles, resulting in the loss of particle diversity.

In addition to the above filtering algorithms, many studies have also proposed other well-known algorithms, including the ensembled Kalman Filter (EnKF) (Hua et al., 2021), the adaptive Kalman Filter (AKF) (Mehra, 1970), and the switched Kalman Filter (SKF) (Wu et al., 2004). However, all methods still inevitably suffer from accuracy and divergence problems.

## 3. SmartFPS architecture

In this section, the general architecture of SmartFPS is presented first. Then, the modules of the network are explained separately. Finally, a multi-task learning-based training method is introduced.

### 3.1. System overview

**Figure 1:**
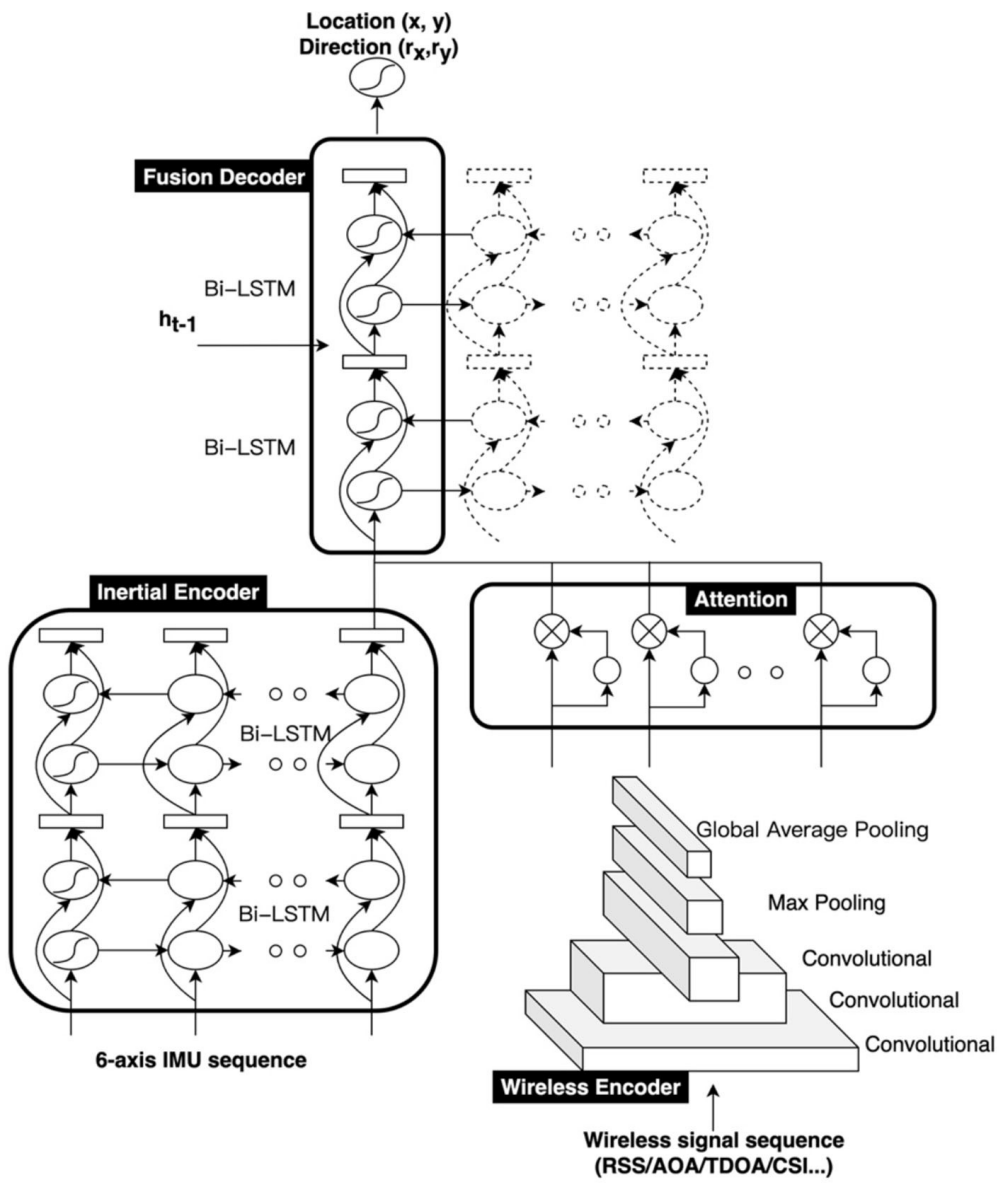
Architecture of SmartFPS.

SmartFPS is an end-to-end trainable neural network for the wireless-inertial fusion navigation system. The inputs of SmartFPS are the inertial signal sequence and the wireless signal sequence. The outputs of SmartFPS are the current pedestrian position and direction. The structure of the fusion positioning system is illustrated in Figure 1. SmartFPS is formed by four modules: (1) inertial encoder, which is a feature extraction module of inertial navigation data based on the LSTM network; (2) wireless encoder, a wireless positioning module based on convolutional neural network (CNN); (3) attention layer; (4) fusion decoder, a fusion positioning module based on LSTM network. The output of the inertial encoder is the latent space tensor of step length and rotation angle, and the output of the wireless encoder module is the extracted wireless signal feature information rather than the final position information. The outputs of the modules pass through the attention layer, then to the fusion localization module. The fusion decoder module uses the hidden state of the last timestep as the initial state and finally estimates the position and direction.

Compared with the filtering fusion positioning system, the fusion positioning system based on the whole network structure has the following advantages:

1) The fusion positioning system with a complete network structure can realize end-to-end computing output, which is easier to train and deploy than the positioning system with networks fused by filtering algorithms. Each positioning network of the network and filtering algorithm need to output noise matrixes during training, and the noise matrix is difficult to represent by individual parameters for complex systems. Therefore, such training and fusion methods are complicated, and the implementation is cumbersome.
2) Compared with the Kalman filter, SmartFPS does not require all noises to be white noise, and there is no need to pre-determine the noise coefficient. It can approximate any white noise or colored noise through data training.
3) The fusion positioning system with a complete network structure is faster than the particle filter, and the time cost of SmartFPS estimation is generally in milliseconds. In contrast, the time cost of the particle filter will be much higher if the number of particles is large. The particle filter will also cause problems such as loss of diversity over time, so parameters such as particle resampling need to be adjusted.

### 3.2. Inertial encoder

The inertial encoder network in the fusion positioning network is an inertial navigation feature extraction network based on the Bi-LSTM network. Its input is the 6-axis accelerometer and gyroscope data in the inertial unit of the smart device, and the signal data set at 1 step can be expressed as (acc_x_, acc_y_, acc_z_, gyro_x_, gyro_y_, gyro_z_). The LSTM network generally processes a time series of data, which in this research scenario are the accelerometer and gyroscope data in a time window. The length of the window can be selected according to different application scenarios. For example, in this study, a time window of 1 s is used by considering the frequency of pedestrian steps.

One primary concern for the inertial encoder is its objective, which represents its physical meaning and may influence the fusion performance. In fact, the inertial encoder derives from former studies of deep learning-based inertial navigation. We carefully select the objective of the inertial encoder among all the related works, such as IONet (Chen et al., 2018) and RoNIN (Herath et al., 2020), and adopt the step length and the rotation angle as IONet did. It is noted that RoNIN assigned the velocity vector (v_x_, v_y_) as the output and achieved excellent performance in accuracy. However, this may cause a problem when the pedestrian keeps walking straightly at a constant speed. In this case, the velocity vector should be possible in all directions so that the trained network is not decoupled from the map. We also found that the trained inertial network with this objective is difficult to fuse with the wireless encoder. The author of RoNIN, although not discussing this point in their work, may have tried to fix this issue by randomly rotating the inertial data for data augmentation before training. However, this method could significantly increase the amount of training data and lower the training speed, which is cumbersome for model transfer learning in a new scenario. Unlike RoNIN, we adopt the step length and the rotation angle, which are completely decoupled from the map.

The objective function of the inertial positioning network is as follows:


$$\begin{array}{l} \underset{\theta }{\min}L(\theta )=\frac{1}{n}\sum_{i=1}^{n}L(x_{i},y_{i};\theta )=\frac{1}{n}\sum_{i=1}^{n}\left(∥\tilde{l}-l{∥}_{2}^{2}+\lambda ∥d\tilde{\varphi }-d\varphi {∥}_{2}^{2}\right)\; \end{array}$$

where *n* represents the number of batch data, θ represents the network parameters, *l* represents the step length, *d*ϕ represents the rotation angle, and λ is the weight of the rotation angle loss. The training target of the entire network includes two parts: step length and rotation angle. Due to the different physical meanings of the two parts, the range of values is also different. For example, the maximum value of the step length is related to the pedestrian step length and the selected time window size, which often remain in the range of [0, 1], and the value of the rotation angle in this study is in the range of (−180°, 180°], so both ranges should be considered to choose the value for λ. In our tests, we set λ to 2.

### 3.3. Wireless encoder

The wireless encoder extracts meaningful characteristics from the wireless signal sequence and maps them to the latent space. Different from previous studies, this study uses dynamically collected wireless time-series signals as input. The input feature is a matrix composed of the signal sequence of the wireless beacons deployed in the entire environment within a time window. The input length is the window size, and the height is the number of wireless beacons. The output 2D position represents the position in the middle of the time window. Both generalization performance and training accuracy can be considered to determine the size of the time window. For example, the use of an excessively long-time window should be avoided. The dynamic acquisition of signal data using a long-time window will cause time dependency, thereby increasing the complexity of the scenario and reducing the generalization performance of the wireless location network. Second, due to the noise of the wireless signal received by the smartphone, if the time window is too short, the network learns the noise signal instead of the signal itself, which will lead to the overfitting of the network. Therefore, the size of the time window should be carefully selected, and the optimal solution can be found by the positioning accuracy of the validation set.

The wireless encoder network is built based on the CNN network. Since the wireless signal is collected dynamically, the dynamic sequence generally considers its timing relationship. However, this network aims to learn the signal itself in the high-noise signal sequence rather than the timing relationship, which can be regarded as a filtering process for high-noise signals. The wireless encoder network mainly includes three convolutional layers, a maximum pooling layer, a global average pooling layer, and two fully connected layers.

The size of the convolution kernel of each layer of the wireless encoder gradually decreases, that is, from global attention to local attention, and the step length also selects different values for tensors with different lengths and widths. The loss function of the wireless location network is as follows:


$$\begin{array}{l} \underset{\theta }{\underset{\theta }{\min}L\left(\theta \right)=\frac{1}{n}\overset{n}{\underset{i=1}{\sum }}L\left(x_{i},y_{i};\theta \right)=\frac{1}{n}\overset{n}{\underset{i=1}{\sum }}\sqrt{{\left(\overset{\sim }{x}-x\right)}^{2}+{\left(\overset{\sim }{y}-y\right)}^{2}}} \end{array}$$

where *x, y* are the two-dimensional position coordinates. It is worth noting that this network uses the “selu” function as the activation function for all CNNs. The “selu” function is expressed as follows:


$$\begin{array}{l} \{\begin{array}{cc} a=\text{λ}z & ,z>0\\ a=\text{λ}\alpha (e^{z}-1) & ,z\leq 0 \end{array}\; \end{array}$$

Compared with the “relu” activation function, “selu” does not have a dead zone. Compared with sigmoid, it also has the advantage that the gradient is not easy to vanish. Compared with the “elu” function, it has the advantage of no parameter selection. The most important role of “selu” is that it can automatically normalize the sample distribution to 0 mean and unit variance, thereby speeding up the network convergence. The function is like the batch normalization layer, but there is no need to increase the network depth. We also found that the “selu” function can improve the training speed.

### 3.4. Attention layer

Asymmetric attention layers are mainly based on the local attention mechanism in deep learning (Vaswani et al., 2017). The attention mechanism is a concept proposed in natural language processing, mainly used to implement a contextual logic between the encoding network and the decoding network. The attention mechanism generally includes three steps: calculating the calibration weight through the hidden state, calculating the softmax weight, and calculating the overall context weight. The concept of the local attention is relative to the global attention. The global attention implements the attention mechanism for all units of the input sequence, while the local attention mechanism implements the attention mechanism for part of the units.

In SmartFPS, the attention mechanism is only used for the output of the wireless encoder network. It is because the hidden state outputs by the wireless encoder network and the inertial encoder network have different physical meanings. Since the output of the inertial encoder network is the hidden state quantity of the last step, it does not have the concept of context itself. Suppose the output of the inertial encoder network adopts the attention mechanism. In that case, it will break the integrity of the part of the hidden state and only input the part of the hidden state sequence into the decoder network. As for the wireless encoder network, due to the different positions of each wireless beacon, when pedestrians are in different positions, the signal strength and stability of each wireless beacon are also different. At the same time, due to factors such as occlusion by walls or multipath effects, it cannot be determined whether to use the signal of the beacon as an input simply by the strength of the signal. Therefore, the attention mechanism can enable the localization network to learn the local characteristics of the environment and realize the regional selection of appropriate wireless signal characteristics, especially when the number of wireless beacons is large. The calculation process of the attention layer is as follows:


$$\begin{array}{lll} u_{t} & = & \tanh\left(Wh_{t}+b\right)\\ \alpha_{t} & = & softmax\left(u_{t}\right)\\ s & = & \overset{M}{\underset{t=1}{\sum }}\alpha_{t}h_{t} \end{array}$$

where *W* refers to the weight of the fully connected layer in the attention layer, *b* is the bias of the fully connected layer, and *h*_*t*_ is the hidden state input to the attention layer. Compared with the global attention mechanism, the asymmetric attention mechanism greatly reduces the number of network parameters, which is of great significance for suppressing model overfitting and improving model training and computing speed.

### 3.5. Fusion decoder

The fusion decoder network is a two-layer LSTM network structure. The hidden state and the unit state of the network are initialized by the state of the previous moment. The input includes the inertial navigation features and the attention-selected wireless positioning signal features, which can be regarded as observations at the current moment. The role of the recurrent neural network here is similar to a filtering process. Unlike filtering algorithms, the noise and the observation are not separated, and the state and the noise feature of the last moment are also used as the hidden state to initialize the network unit. State quantities, observations, and system processes and noise are coupled through the network, thereby eliminating the tedious process of determining system noise values. Especially for the wireless location system based on fingerprint location, the noise at different locations is more difficult to quantify. The hidden state quantity output by the fusion decoder network will be further connected to two fully connected layers. The output of the last fully connected layer is the two-dimensional position coordinate vector (x, y) and direction vector (r_x_, r_y_) of the pedestrian. The target is set as the root mean square error of the two-dimensional coordinate vector and the direction vector, with a scale factor of κ.

The final objective function of the fusion decoder network is as follows:


$$\begin{array}{l} \begin{array}{l} \underset{\theta }{\min}L(\theta )=\sum^{\text{​}}L(x,y;\theta )=\sum^{\text{​}}∥\tilde{d}-d{∥}_{2}^{2}+\kappa ∥\tilde{r}-r{∥}_{2}^{2} \end{array} \end{array}$$

where θ is the network parameter, *d* represents the two-dimensional position vector (*x, y*), *r* represents the direction vector (r_x_, r_y_).

### 3.6. Training method based on multi-task learning

Since SmartFPS is a large network consisting of several modules, the training procedure should be carefully treated. Since the encoders have different objectives, training them together may corrupt their physical meanings, causing the whole network to collapse. Training them individually and freezing their weights when training the fusion decoder is a good option but cumbersome. Therefore, we proposed a multi-task learning strategy to solve this problem. Multi-task learning (Caruana, 1997) is a branch of deep learning that aims to improve learning efficiency by exploiting the similarity between different tasks to solve multiple different tasks simultaneously. Here the loss of the fusion decoder and the loss of each encoder can be trained simultaneously. The objective of our multi-task learning network is:


$$\begin{array}{l} \underset{\theta }{\min}L\left(\theta \right)=\underset{\theta }{\min}\left(L_{fusion}\left(\theta \right)+{\text{λ}}_{1}L_{ins}\left(\theta \right)+{\text{λ}}_{2}L_{wireless}\left(\theta \right)\right) \end{array}$$

Although multi-task learning is generally used to improve learning efficiency, it can protect the integrity of the output features of the intermediate layer, and the fixed output positions can retain the physical meaning of the output of each sub-network.

## 4. SmartFPS transfer learning

Since the environmental factors and device parameters are often different from the training data in practical scenarios, trained models often perform poorly. In this section, a transfer learning method is proposed to solve this problem based on the generative adversarial network mechanism. In this section, the attack factors that may cause the trained models to fail are discussed. Next, the proposed transfer learning method is explained in detail.

### 4.1. Attack factors

**Figure 2:**
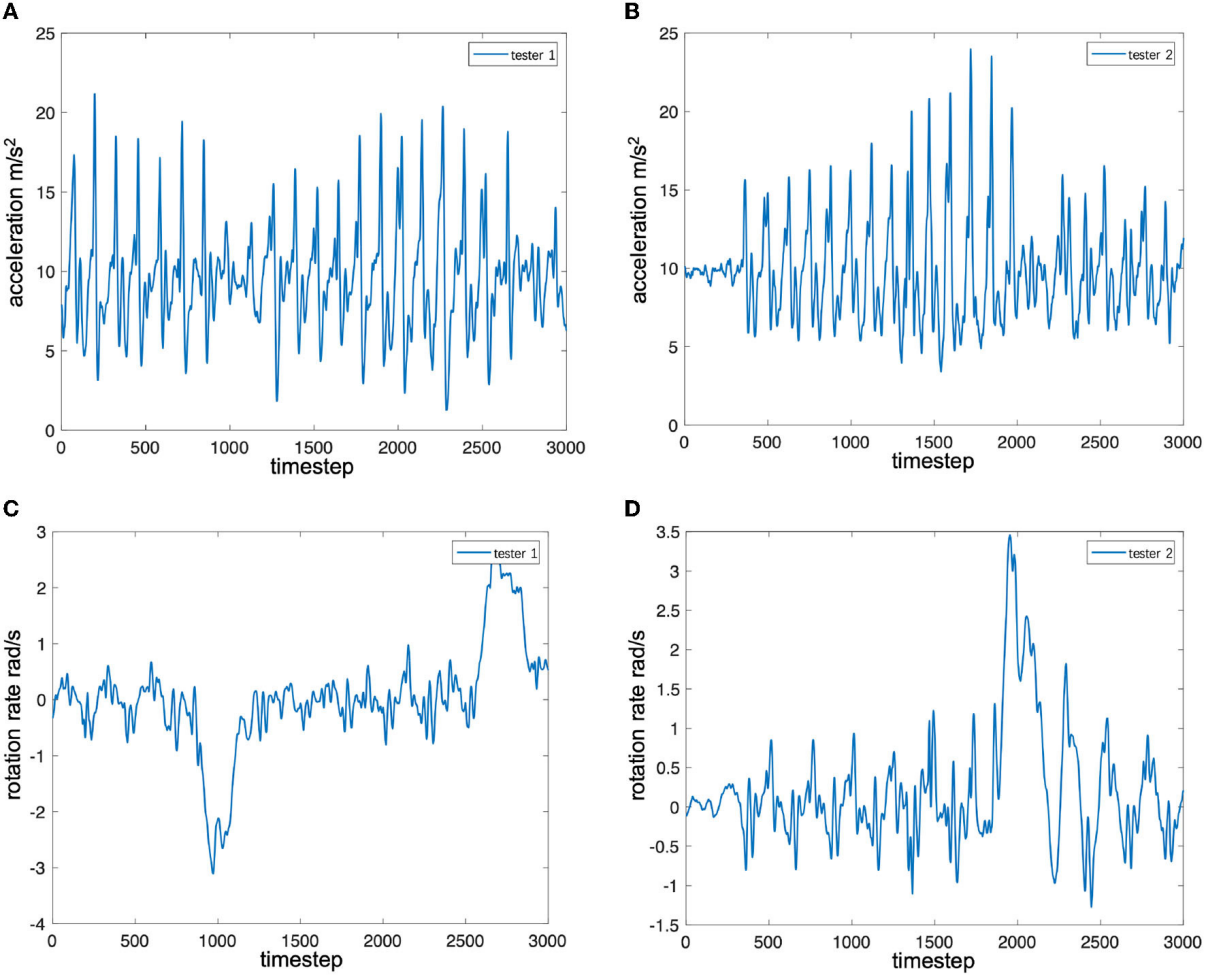
Signal sequence of inertial sensors. **(A)** Accelerator along z-axis for tester 1 (1.76 m). **(B)** Accelerator along z-axis for tester 2 (1.63 m). **(C)** Gyroscope along z-axis for tester 1 (1.76 m). **(D)** Gyroscope along z-axis for tester 2 (1.63 m).

To analyze the influence of different pedestrians on the characteristics of the inertial data, two testers collected inertial data by carrying the same device and walking back and forth along the same straight-line path. The pose of the smartphone in hand was similar to that of the training set. Figures 2A–D shows the time series of the accelerometer and gyroscope data collected by two testers (person 1 1.76 m and person 2 1.63 m). From Figures 2A, B, it can be found that the peak-to-peak ratio of the z-axis acceleration of tester 1 is greater than that of tester 2, that is, the mobile phone swings up and down with greater force. In addition, it can be found that there are frequent sub-peaks near the peak in tester 2's data, which is also caused by the tester's walking habit. It can be observed from Figures 2C, D that the peak-to-peak value of the gyroscope data of tester 1 is smaller than that of tester 2. Considering these attack factors, most of the pedestrian inertial navigation positioning research needs to add the analysis of pedestrian motion characteristics to the system. However, it is difficult to accurately model the diversity of the characteristics and changes of different pedestrian movements, so it is difficult to accurately track the position of pedestrians by relying solely on the pedestrian inertial navigation system.

**Figure 3:**
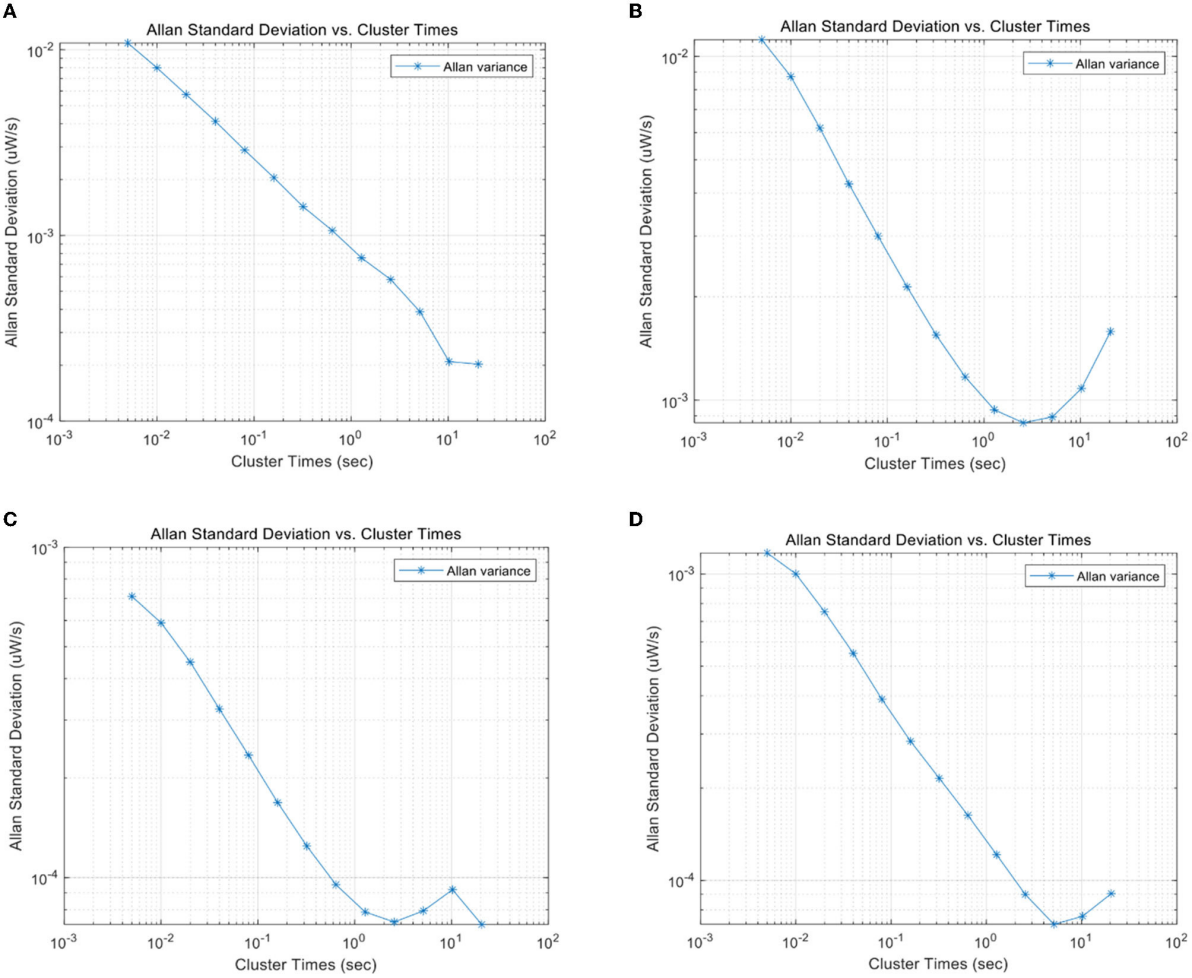
**(A)** Allan variance of Mi 6's accelerator along x-axis. **(B)** Allan variance of ZTE A2017's accelerator along x-axis. **(C)** Allan variance of Mi 6's gyroscope along x-axis. **(D)** Allan variance of ZTE A2017's gyroscope along x-axis.

In addition to different pedestrian motions, different devices also cause the difference in data characteristics. Since the calibration methods can solve the problems such as bias and drift, we only consider the noise characteristics of the inertial device itself of different devices. To verify the difference in noise characteristics of inertial systems of different devices, two devices (Xiaomi Mi6 and ZTE A2017) are used to collect inertial data in a horizontal stationary state. Allan variance was used to analyze the noise components in the inertial data. The Allan variance curves of the accelerometer and gyroscope sequences of the two devices in the static state are shown in Figures 3A–D. From Figures 3A, B, it can be observed that the major noise component of the accelerometer of Mi6 and A2017 within 1 s is the Gaussian white noise, but the coefficients are quite different. For A2017, there are colored noise components with a small magnitude near 1 s. From Figures 3C, D, white noise is detected by both gyroscopes' sequences but with different coefficients. Therefore, the noise characteristics of inertial sensors are quite different between different devices.

**Figure 4:**
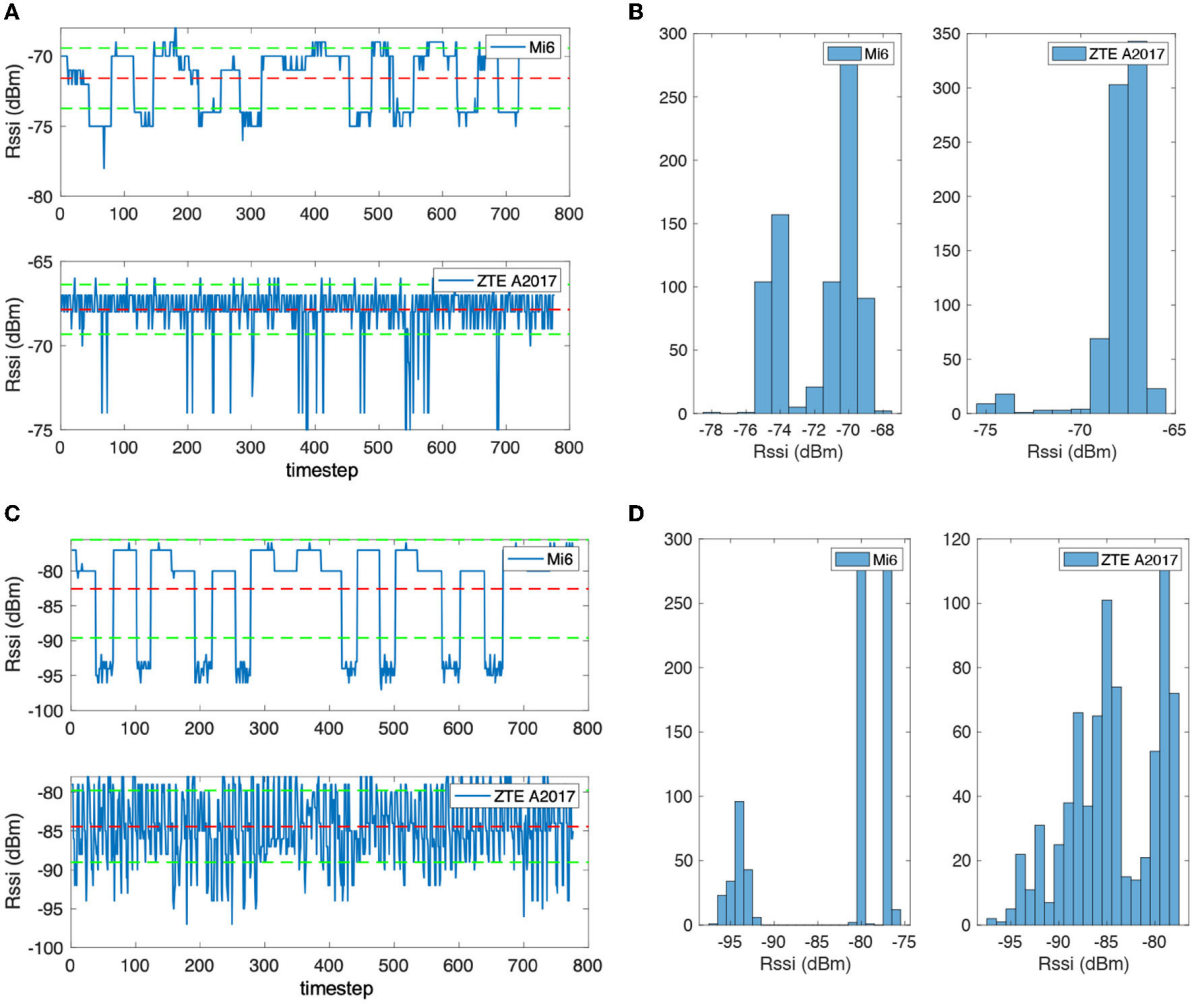
**(A)** The received signal strength sequence of a Bluetooth beacon with 0.6 m distance. **(B)** The received signal strength sequence of a Bluetooth beacon with 1.5 m distance. **(C)** The received signal strength distribution of a Bluetooth beacon with 0.6 m distance. **(D)** The received signal strength distribution of a Bluetooth beacon with 1.5 m distance.

There are also differences in the wireless signal receivers of different mobile phone devices. To verify this problem, two mobile phones were placed horizontally on the table and kept the same distance from the Bluetooth beacon to collect RSS data. Figures 4A–D are the RSS sequence when the distance from the Bluetooth beacon is 0.6 and 1.5 m. Figures 4A, B show the Bluetooth RSS data of the two devices at 0.6 m. Figure 4A is the signal timing diagram, in which the middle-dotted line represents the mean value, and the upper and lower dotted lines represent the mean value plus or minus the standard deviation. Figure 4B shows the statistical histograms of different signal strength values. It can be observed that the sequential noise of the two devices is quite different, and the high noise is induced in the A2017 signal. In addition, although the distance between the two devices and the Bluetooth beacon is the same, differences can also be found in the RSS values. The average value of the Mi6 is −72 dBm, and the A2017 is −68 dBm. From the histogram, there are two obvious peaks in the histogram of Mi6, and the amplitude values are obviously discrete. The A2017 noise frequency is higher, but the change of the amplitude is smaller. Many outliers can be found in the timing diagram of the A2017 signal. Figures 4C, D show the RSS value at 1.5 m. It can be seen from Figure 4C that the signal of A2017 still has the characteristics of high-frequency noise. The histogram in Figure 4D shows that the two peaks of the Mi6's Bluetooth signal are farther apart, and the A2017 also shows the characteristics of the two peaks.

In general, there are irregular differences in the amplitude and noise characteristics of inertial and wireless signal data. Therefore, it is necessary to consider how to enhance the generalization performance of SmartFPS under those differences.

### 4.2. Transfer learning based on GAN

The transfer learning algorithm of SmartFPS aims at training the first 3-layer CNN network layer in the wireless positioning network and the first 2-layer LSTM network in the inertial positioning network. Since the transfer learning methods of the two networks are similar, only the transfer learning method of the wireless positioning network is explained here.

The wireless encoder aims to map the features of the wireless signal data sequence, so the transfer learning algorithm aims to train those layers and substitute the trained weights into the fusion positioning network. The transfer learning structure of the wireless positioning network is mainly based on the GAN network, a transfer learning strategy known for its success in image processing. GAN is composed of a generator and a discriminator. The generator's role is to produce a fake wireless sequence similar to the target domain according to the wireless positioning features of the source domain. The discriminator will judge whether the generator produces sequences similar to the target domain. Therefore, the generator will improve its performance with each training epoch, and eventually, the discriminator will not be able to make the right judgment.

We also add a cycle-consistent loss to the network so that the generator network will include a source and a target domain generator. These two parts share one unique encoder. According to cycleGAN, the source domain generator network needs to restore the sequence of fake target domain signals generated by the target domain generator (the fake target domain signals are also derived from the source domain data) to the source domain data. Similarly, the target domain generator network needs to restore the sequence of fake target domain signals generated by the source domain generator (fake source domain signals are generated from target domain data) to target domain data. Such a mechanism makes GAN less influenced by the generator network when the source and target data labels are poorly matched. In addition, a new loss function, identity loss, is often introduced when applying cycle-consistent loss. This mechanism means that data in the same domain should maintain the same data through the same domain generator network.

We also introduce the reconstruction network in the network. The reconstruction network is a CNN structure, and its role is to restore the original signal using the encoder output features. Unlike the cycle-consistency loss mechanism, the reconstruction network is unique. Although the recovered signal will not be the same as the original signal, it can preserve the signal integrity as much as possible from the common characteristics of the source and target domains. This part is also to protect the encoder from being unduly influenced by the generative network. Since the GAN network needs to train the generator and the discriminator separately, we also divide the training of the inertial positioning network transfer learning network into two steps: (1) training the generator targets and (2) training the discriminator targets.

**Figure 5:**
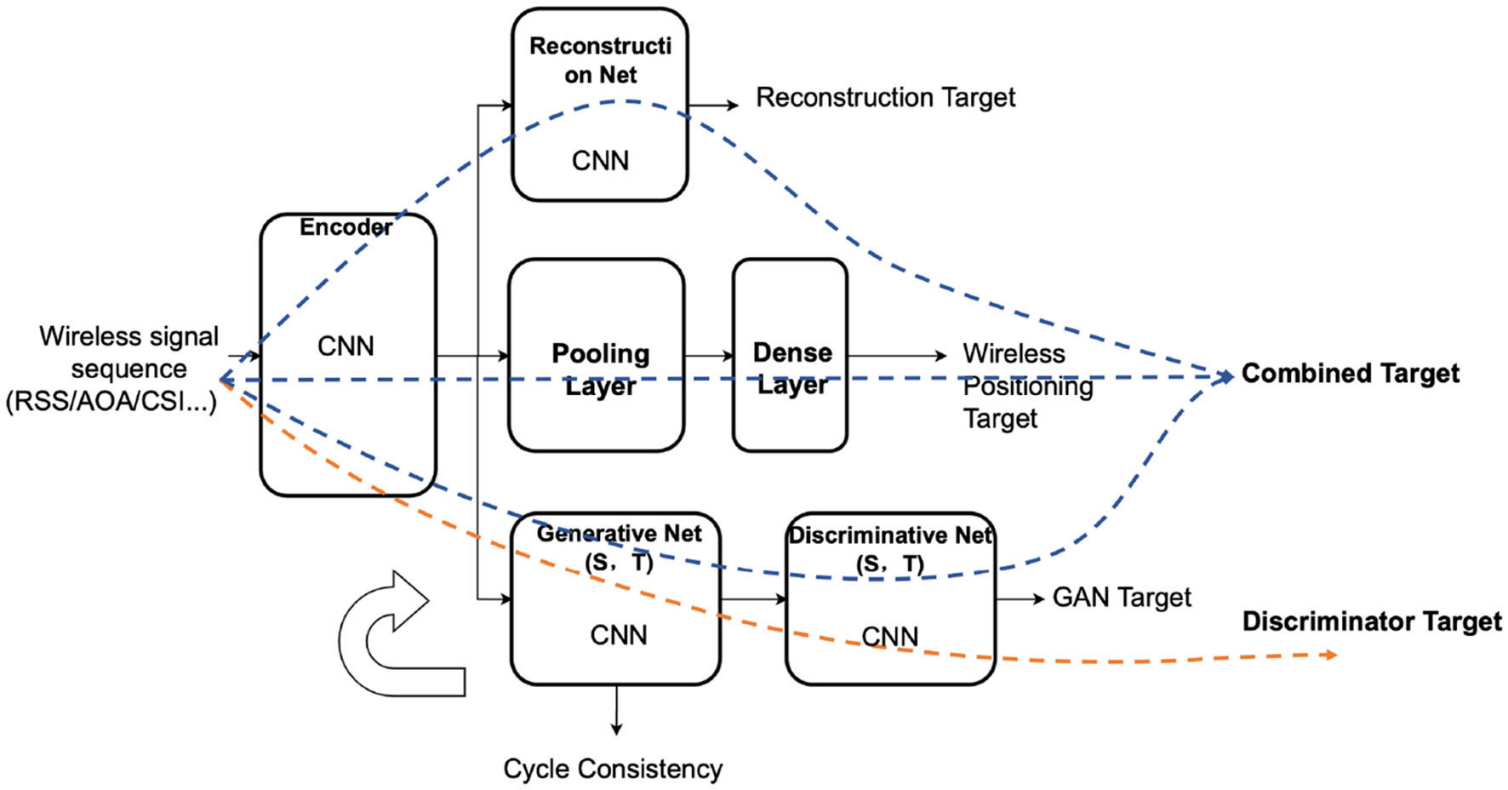
Transfer learning structure of wireless positioning encoder in SmartFPS.

The network forward propagation routes of the two targets are illustrated in Figure 5. The generator and the encoder are trained by the wireless positioning loss, the reconstruction loss, the consistency loss, and the cycle consistency loss. Different weight factors combine those losses into one single target. The combined loss function is (take the source domain as an example):


$$\begin{array}{l} L_{c,S}=L_{gan}+{\text{λ}}_{1}L_{cycle}+{\text{λ}}_{2}L_{identity}+{\text{λ}}_{3}L_{pred}+{\text{λ}}_{4}L_{recon} \end{array}$$

The training procedure of wireless positioning encoder transfer learning is similar to training GAN. It is important to train only the generator or the discriminator alone while freezing the weights of the other network. However, the other goals can be trained together. The training procedure is listed in Table 1. The transfer learning structure of the inertial encoder is similar to the wireless positioning encoder, and only the CNN network should be replaced by LSTM. After training the wireless positioning encoder and inertial encoder separately, the weights of each encoder can be used in the fusion network to accomplish the transfer learning target.

**Table 1:** Algorithm 1: Training procedure of wireless positioning encoder transfer learning.

**Training procedure of wireless positioning encoder transfer learning**		
1.	Get weights of the wireless encoder of SmartFPS.	
2.	Initialize weights of wireless encoder in the transfer learning network and freeze weights of pooling layer and dense layer.	
3.	For t = 1 < Number of iterations do:	
4.	Sampling *N* labeled data from source domain;	
5.	Sampling *N* unlabeled data from target domain;	
6.	Generate target domain fake data from source domain data *G_*S*_*;	
7.	Generates source domain fake data from target domain data *G_*T*_*;	
8.	Freeze the *D_*S*_* weights and update combined network weights according to source domain combined target: $\begin{array}{l} \nabla_{\theta_{F},\theta_{G_{S}},\theta_{C}}\sum_{i=1..N}L_{gan}({\overset{˘}{D}}_{S}(G_{S}(F(x_{T,i},\theta_{F}),\theta_{G_{s}}),\theta_{D_{s}}),y_{real})+\;\\ \lambda_{1}(L_{cycle}\left({\overset{˘}{G}}_{T}\left(F\left(G_{S}\left(F\left(x_{T,i},\theta_{F}\right),\theta_{G_{S}}\right),\theta_{F}\right),\theta_{G_{T}}\right),x_{T,i}\right)+\;\\ \;L_{cycle}\left(G_{S}\left(F\left({\overset{˘}{G}}_{T}\left(F\left(x_{S,i},\theta_{F}\right),\theta_{G_{T}}\right),\theta_{F}\right),\theta_{G_{S}}\right),x_{S,i}\right))+\;\\ \lambda_{2}L_{identity}(G_{S}(F(x_{S,i}),\theta_{G_{s}}),x_{S,i})+\lambda_{3}L_{pred}(\overset{˘}{R}(F(x_{S,i},\theta_{F}),\theta_{R}),y_{S,i})+\;\\ \lambda_{4}(L_{recon}(C(F(x_{S,i},\theta_{F}),\theta_{C}),x_{S,i})+L_{recon}(C(F(x_{T,i},\theta_{F}),\theta_{C}),x_{T,i})) \end{array}$	
9.	Freeze wireless encoder, generator, and update discriminator weights according to source domain discriminator target: $\begin{array}{c} \nabla_{\theta_{D_{S}}}\sum_{i=1..N}(L_{gan}\left(D_{S}\left(x_{S,i},\theta_{D_{S}}\right),y_{real}\right)+\\ L_{gan}(D_{S}({\overset{˘}{G}}_{S}(\overset{˘}{F}(x_{T,i},\theta_{F}),\theta_{G_{S}}),\theta_{D_{S}}),y_{fake})) \end{array}$	
10.	Freeze *D_*T*_* weights, and update combined network weights according to target domain combined target: $\begin{array}{l} \;\nabla_{\theta_{F},\theta_{\theta_{T}},\theta_{C}}\sum_{i=1..N}L_{gan}({\overset{˘}{D}}_{T}(G_{T}(F(x_{S,i},\theta_{F}),\theta_{G_{T}}),\theta_{D_{T}}),y_{real})+\\ \;\lambda_{1}(L_{cycle}\left({\overset{˘}{G}}_{S}\left(F\left(G_{T}\left(F\left(x_{S,i},\theta_{F}\right),\theta_{G_{T}}\right),\theta_{S}\right),\theta_{G_{S}}\right),x_{S,i}\right)+\;\\ \;L_{cycle}\left(G_{T}\left(F\left({\overset{˘}{G}}_{S}\left(F\left(x_{T,i},\theta_{F}\right),\theta_{G_{S}}\right),\theta_{F}\right),\theta_{G_{T}}\right),x_{T,i}\right))+\;\\ \lambda_{2}L_{identity}\left(G_{T}\left(F\left(x_{T,i}\right),\theta_{G_{T}}\right),x_{T,i}\right)+\;\lambda_{3}L_{pred}\left(\overset{˘}{R}\left(F\left(x_{S,i},\theta_{F}\right),\theta_{R}\right),y_{S,i}\right)+\;\\ \;\lambda_{4}(L_{recon}(C(F(x_{T,i},\theta_{F}),\theta_{C}),x_{T,i})+L_{recon}(C(F(x_{S,i},\theta_{F}),\theta_{C}),x_{S,i})) \end{array}$	
11.	Freeze wireless encoder, generator, and update discriminator network weights according to target domain discriminator target: $\begin{array}{c} \nabla_{\theta_{D_{T}}}\sum_{i=1..N}(L_{gan}(D_{T}(x_{T,i},\theta_{D_{T}}),y_{real})+\\ L_{gan}(D_{T}({\overset{˘}{G}}_{T}(\overset{˘}{F}(x_{S,i},\theta_{F}),\theta_{G_{T}}),\theta_{D_{T}}),y_{fake})) \end{array}$	
12.	End	

## 5. Results

### 5.1. Experiment settings

**Figure 6:**
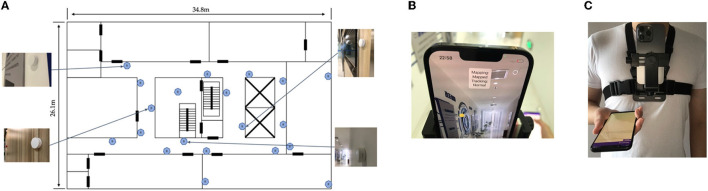
**(A)** Experiment testbed. **(B)** Lidar slam on iPhone. **(C)** Device setup on body.

The experiments were conducted in the corridor area plus a whole laboratory area on one floor of the Chuangzhi Building, Nanjing, China, as shown in Figure 6A. The test area is about 20 m long and 30 m wide. A total of 20 Bluetooth beacons were deployed in the venue, and the distance between each Bluetooth beacon was 5–8m, of which No. 17–20 Bluetooth beacons were deployed inside the laboratory. The experimental site includes the stair hall and the elevator hall that may block the Bluetooth signals, so there are generally 3 or 4 line-of-sight Bluetooth signals received by smartphones during positioning. The Bluetooth beacon was the E5 model of Yunli Physics. We changed the broadcast period from 500 ms (default) to 100 ms to increase the training data density. The broadcast power of the product is 0 dBm by default with a coverage radius of 50 m, and we adjusted it to −8 dBm with a coverage radius of 22 m. The beacons were stuck on the wall 1.5 m above the ground.

The training and test data were collected with the android phone facing the user by assuming that the user should keep an eye on the screen of the smartphone during positioning. The smartphone was swinging along with the user's arm. To collect training data of accelerometer, gyroscope and Bluetooth received signal strength data, Android phones, including Xiaomi Mi6 and Vivo x70 pro, were selected in this study. The sampling frequency of the inertial sensor is 200 Hz, and the sampling frequency of the Bluetooth signal is set to the highest in the system.

In IONet, the Vicon system was used to track the position of the mobile phone. However, considering the difficulty of deploying the system in a large scene, we implemented a slam system to acquire positions. Different from RoNIN, where the Zenfone AR mobile phone used in it completely relies on the rear camera to calculate the slam, our research firstly uses the Lidar visual slam system of the iPhone, and the positioning accuracy is much higher than that of the Android tango system. Based on the ARkit library of iOS 15, this research implements an application that reads and saves the pose of the mobile phone in real time. At the same time, the application will also read the real-time inertial navigation data of the iPhone to facilitate time calibration with the Android data. Figure 6B shows the actual operation of the Lidar vision SLAM positioning label collection platform based on the iOS ARkit. The position sampling frequency of the slam system is 20 Hz. To verify the positioning accuracy of the system, we selected a rectangular trajectory as a reference according to the texture of the floor tiles in the experimental scene and carried out multiple continuous collections of 1-h slam, walking freely throughout the whole process, and walking according to the reference line every 15 min. By comparing the walking trajectories of the reference routes in different periods, we found that the cumulative drift error of the iPhone's Lidar SLAM system does not exceed 20 cm in 1 h, which is a significant improvement compared to the Tango system mentioned in RoNIN, which has an error of <30 cm in 15 min. The SLAM phone was attached to the chest of the user's body, as shown in Figure 6C. Therefore, compared with the Vicon system, which directly locates the phone's position, there was uncertain bias in the position and the direction of the Android phones. This may compromise the accuracy of the estimated step length and rotation angle, which are the essentials of the inertial encoder's objective. However, the fusion system can lower this influence on the final objective.

The preprocessing procedure was similar to the work (Herath et al., 2020), including spatial and time calibration. The Bluetooth signal sequence and the SLAM's positions should also be down-sampled due to their uneven sampling frequencies. In this study, the inputs of both encoders are 1 s of the signal sequence. The Bluetooth RSS value is chosen as the input of the wireless encoder and down-sampled to 100 Hz. The input of the inertial encoder is 200 steps of inertial data. Hyperparameters of SmartFPS are summarized in Table 2, where “IE” refers to the inertial encoder, “WE” refers to the wireless encoder, “AT” refers to the attention layer, and “FD” refers to the fusion decoder.

**Table 2:** Parameter settings of SmartFPS.

**Parameter**	**Value**
Layers	IE: Input1 (200, 6), WE: Input2 (100, 20) IE: Bi-LSTM (128) IE: Dropout (0.25) IE: Bi-LSTM (128) IE: Dropout (0.25) WE: Conv (16, kernel: 7, stride: (2,2), activation: “selu”) WE: Conv (32, kernel: 5, stride: (2,2), activation: “selu”) WE: Conv (64, kernel: 3, stride: (5,1), activation: “selu”) WE: MaxPooling (2) WE (O_WE_): GlobalAveragePooling () AT: Dense(“selu”)* O_WE_ FD: Bi-LSTM() FD: Dropout (0.2) FD: Dense (3), Dense (3)
Optimizer	“Adam” *lr* = 0.0008 beta1 = 0.9 beta2 = 0.999 epsilon = 1e-07
Batchsize	512
Max epoch	100

### 5.2. SmartFPS tests

**Figure 7:**
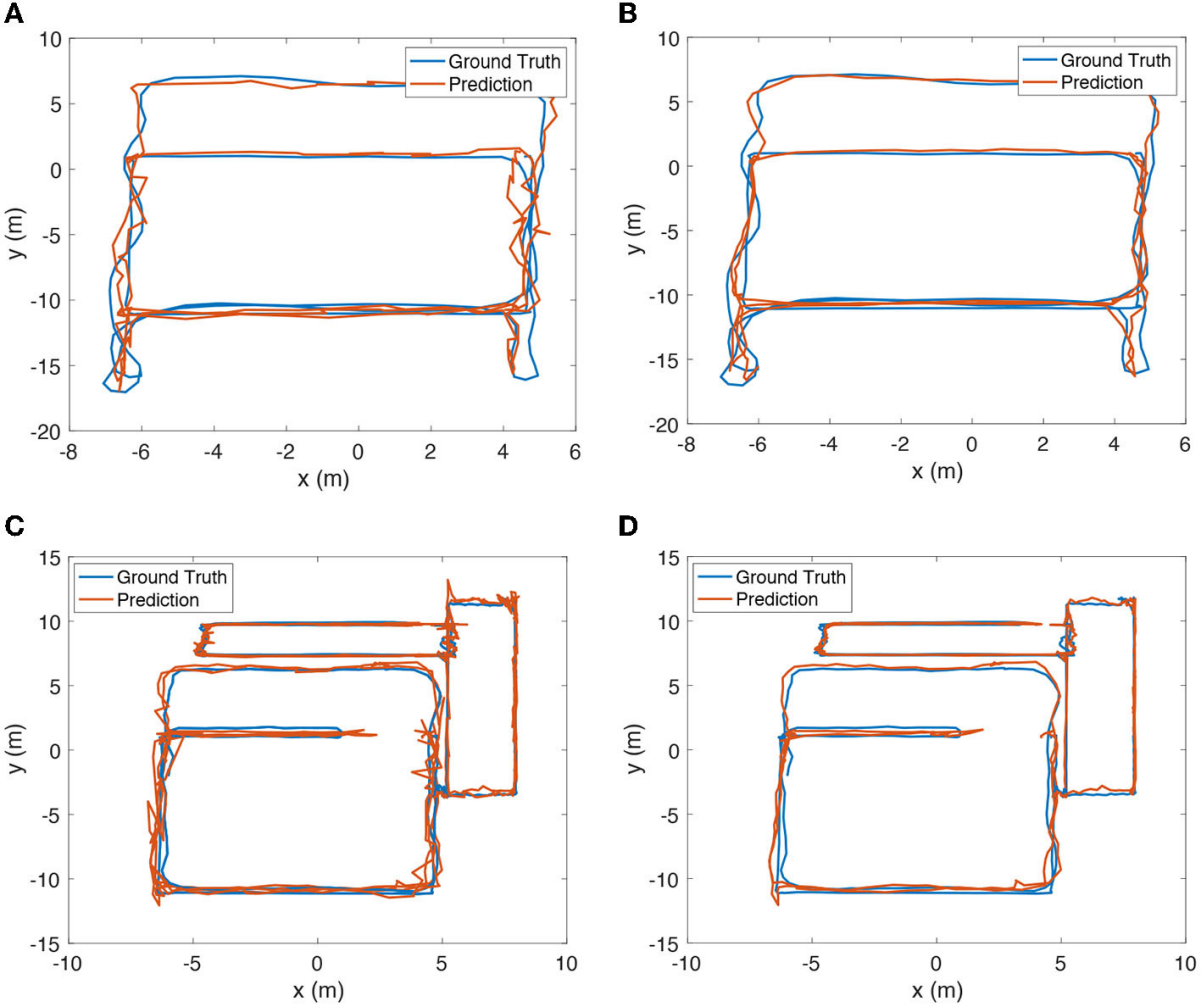
**(A)** Estimated trajectory of test set 1 by wireless positioning encoder of SmartFPS. **(B)** Estimated trajectory of test set 1 by SmartFPS. **(C)** Estimated trajectory of test set 2 by wireless positioning encoder of SmartFPS. **(D)** Estimated trajectory of test set 2 by SmartFPS.

The positioning results of the wireless encoder and SmartFPS of a set of test trajectories are depicted in Figure 7. Smoother trajectories were predicted by SmartFPS than the wireless encoder, indicating that SmartFPS successfully reserve the physical meaning of the inertial encoder, which is the relative translation from restricted step length and rotation angle. On test set 1, the average positioning error of the wireless encoder was 0.695 m, while SmartFPS achieved 0.467 m. The error of the wireless encoder and SmartFPS are 0.760 and 0.583 m on test set 2. On all test sets, the average positioning error of the wireless encoder was 0.739 m, while SmartFPS achieved 0.506 m.

To compare the performance of SmartFPS and filter fusion algorithms, two types of fusion systems based on extended Kalman filter and particle filter were tested. The filters were used to fuse the outputs of the pre-trained wireless encoder and inertial encoder. Among them, for the extended Kalman filter, the variance matrix of the observed amount of the Bluetooth estimated position is determined by the pre-trained network using the variance value of the error between the prediction result of the training set and the actual value. The relationship between the two-dimensional position vector and the rotation angle is:


$$\begin{array}{l} \{\begin{array}{c} x_{k+1}=x_{k}+l_{k}\cos\varphi_{k}\\ y_{k+1}=y_{k}+l_{k}\sin\varphi_{k} \end{array} \end{array}$$

Then, the process equation for the state is derived from the above formula:


$$\begin{array}{l} \{\begin{array}{c} \begin{array}{c} dx_{k+1}=dx_{k}+dl_{k}\cos\varphi_{k}-l_{k}d\varphi_{k}\sin\varphi_{k}+dw_{x}\\ dy_{k+1}=dy_{k}+dl_{k}\sin\varphi_{k}+l_{k}d\varphi_{k}\cos\varphi_{k}+dw_{y} \end{array}\\ \begin{array}{c} dl_{k+1}=dl_{k}+dw_{l}\\ d\varphi_{k+1}=d\varphi_{k}+dw_{\varphi } \end{array} \end{array} \end{array}$$

Therefore, the process matrix is:


$$\begin{array}{l} {\text{Φ}}_{k}=\left[\begin{array}{cccc} 1 & 0 & \cos\varphi_{k} & -l_{k}\sin\varphi_{k}\\ 0 & 1 & \sin\varphi_{k} & l_{k}\cos\varphi_{k}\\ 0 & 0 & 1 & 0\\ 0 & 0 & 0 & 1 \end{array}\right] \end{array}$$

The observation equation is:


$$\begin{array}{l} Z={\left[dx_{k},dy_{k}\right]}^{T}=\left[x_{ble,k}-x_{pred,k},y_{ble,k}-y_{pred,k}\right]\; \end{array}$$

Therefore, the observation matrix is:


$$\begin{array}{l} H_{k}=\left[\begin{array}{c} 1\;0\;0\;0\\ 0\;1\;0\;0 \end{array}\right] \end{array}$$

The process noise matrix is:


$$\begin{array}{l} Q_{k}=\left[\begin{array}{cccc} \delta_{x}^{2} & 0 & 0 & 0\\ 0 & \delta_{y}^{2} & 0 & 0\\ 0 & 0 & \delta_{l}^{2} & 0\\ 0 & 0 & 0 & \delta_{\varphi }^{2} \end{array}\right] \end{array}$$

The observation noise matrix is:


$$\begin{array}{l} R_{k}=\left[\begin{array}{cccc} R_{x} & 0 & 0 & 0\\ 0 & R_{y} & 0 & 0 \end{array}\right] \end{array}$$

The implementation of the particle filter system is mainly based on the process equation, which will not be repeated here. We set the number of particles to 700, as suggested by Chen et al. (2022). It is worth noting that how to set the position state noise in the process noise greatly influences the positioning accuracy of the filter-based system. However, the effects of positional uncertainty are difficult to model. To determine the process noise coefficient of each step, the variance of the network output results before and after adding noise several times is mainly used as the noise coefficient of the position. Based on this method, this experiment can further reflect the performance advantage of the network fusion algorithm compared to the filtering algorithm.

**Figure 8:**
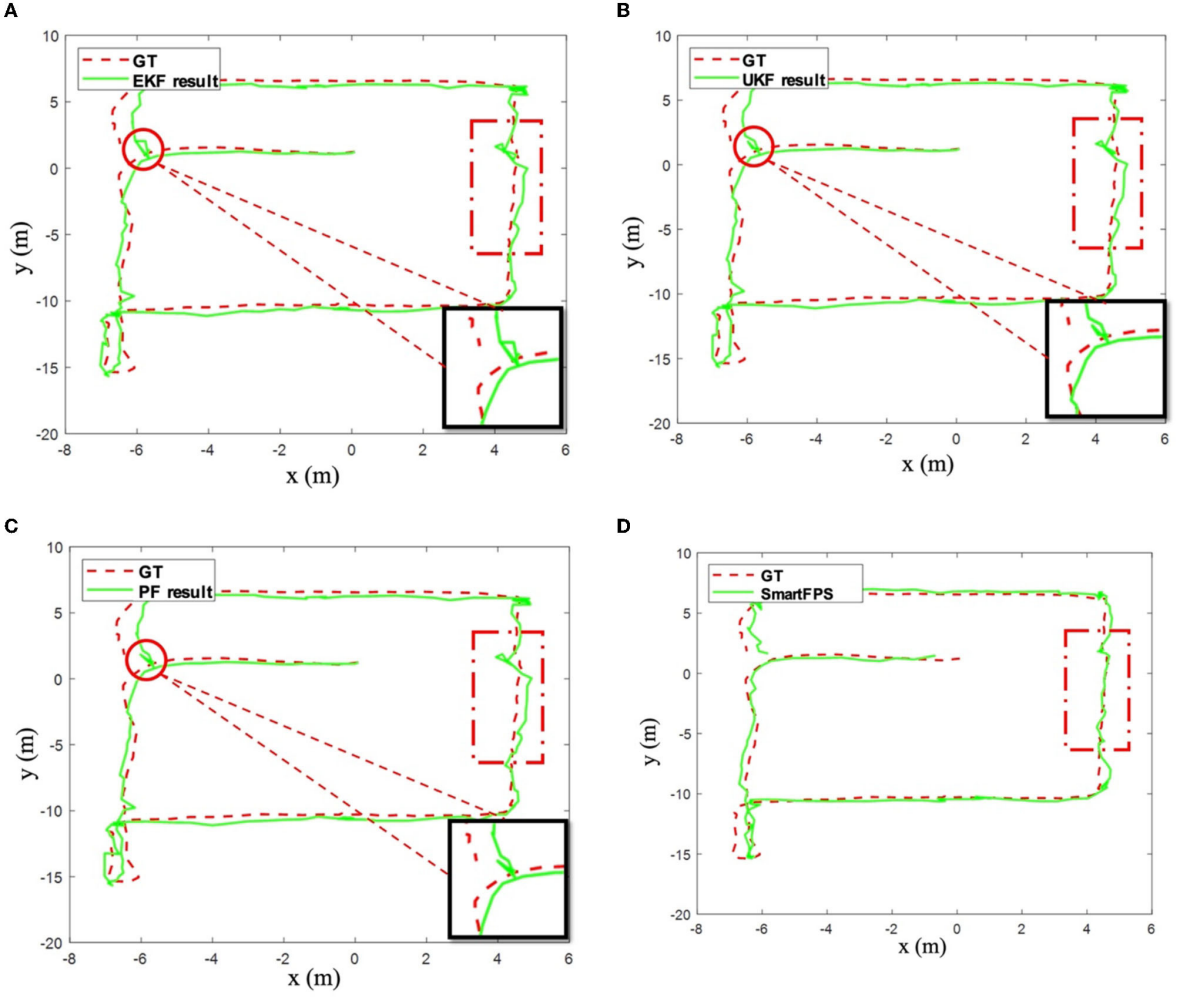
**(A)** Testset 1 result by EKF. **(B)** Testset 1 result by UKF. **(C)** Testset 1 result by PF. **(D)** Testset 1 result by SmartFPS.

The positioning results of the implemented filtering algorithm and SmartFPS are shown in Figures 8A–D. The positioning trajectories of EKF and UKF are highly similar (refer to the circle in the figure and the enlarged part). This is because the same process noise and observation noise parameters are used. The test results of PF are generally better than other filtering algorithms. The predicted trajectory of SmartFPS is closest to the ground truth (refer to the part marked by the dotted solid line in the figure). It is noted that in some areas, the filter-based algorithm shows large fluctuations, while the fusion network is more stable and smoother.

**Figure 9:**
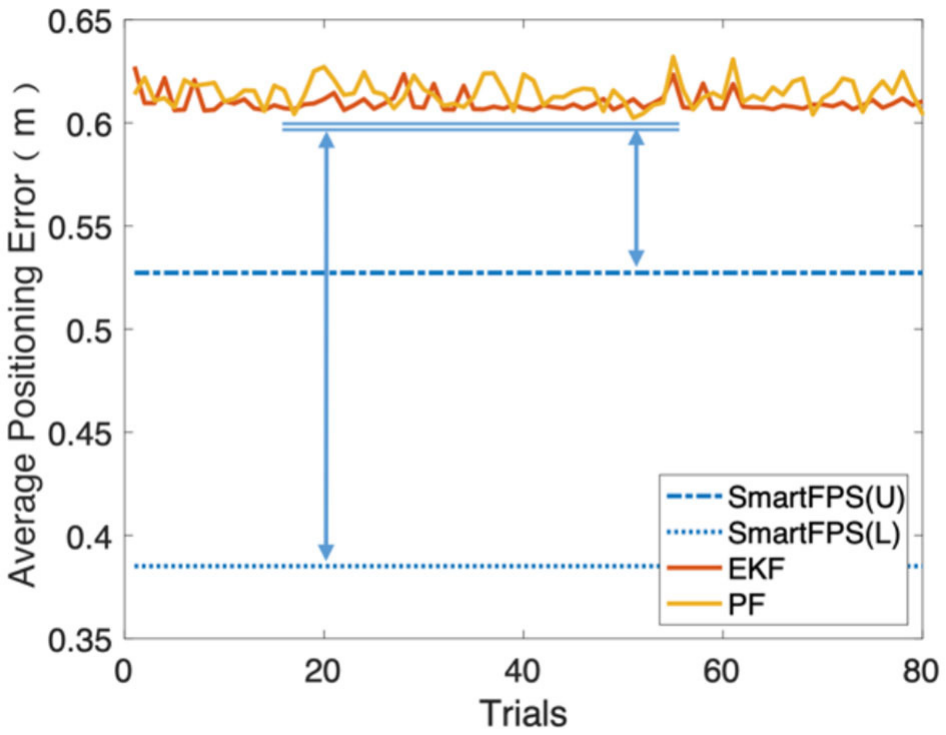
Gap between the positioning errors of filter methods and SmartFPS.

In addition, during the test, we found that the process noise and observation noise coefficients calculated by the training set in the filter-based algorithm are not optimal. Besides, applying process noise and observation noise coefficients different from the statistical results greatly impacts the final positioning accuracy. Based on the statistical results, a global search strategy was used to search for the optimal solution of the process noise coefficient of the EKF and PF algorithms, and up to 5% of the increase or decrease was randomly added to the optimal value. The test results are shown in Figure 9. Compared to the filter fusion algorithms, which adopt the optimal parameters, there is still a significant gap between the positioning accuracy of filter-based fusion and SmartFPS. It can be seen from Figure 9 that the lower bound (best in all tests) of the positioning error of SmartFPS was improved by 36.5%. For the upper bound, the accuracy was also improved by 13%.

The computation time of filter methods, SmartFPS, and sub-networks of SmartFPS are summarized in Table 3. Since the filter-based methods fuse the output of each sub-network trained by individual objectives, the training time should be summed up to be equal for all filter methods. As can be seen from Table 3, the training time for one epoch of SmartFPS was 4.804 s, which is less than the sum of sub-networks. The reason is that SmartFPS was trained by multiple objectives simultaneously so that the parallel computing power of GPU can be maximized. By setting the early stopping ratio to 0.5% and its patience to 3 epochs, the average training epoch for the inertial network was 21, 8 for the wireless network, and 37 for SmartFPS. The average training time only accounts for the computational cost during the offline stage, and the average delay is essential for real-time pedestrian navigation. To output a location at one timestep, the filter methods should first acquire the outputs from sub-networks. Therefore, the computational time consists of the delay from each sub-network. As can be seen from Table 3, the difference in average delays of filter methods and SmartFPS is relatively small. It is noted that during the test stage, the batch size for SmartFPS was set to 1. As a result, the average delay of SmartFPS was no less than the sum of the delay of sub-networks. It can also be found that the fusion decoder almost has no delay compared to encoders, and even the filter methods were slower by milliseconds. Overall, the average delay of SmartFPS is satisfying for real-time navigation.

**Table 3:** Computation performance of filter methods and SmartFPS.

**Methods**	**Average training time (s/epoch)**	**Average delay (s)**
Inertial network	3.116	0.058
Wireless network	3.520	0.015
EKF fusion	6.636	0.074
PF fusion (700 particles)	6.636	0.075
PF fusion (2,000 particles)	6.636	0.076
SmartFPS	4.804	0.073
Inertial network transfer	2.213	-
Wireless network transfer	2.418	-

### 5.3. SmartFPS transfer learning tests

**Figure 10:**
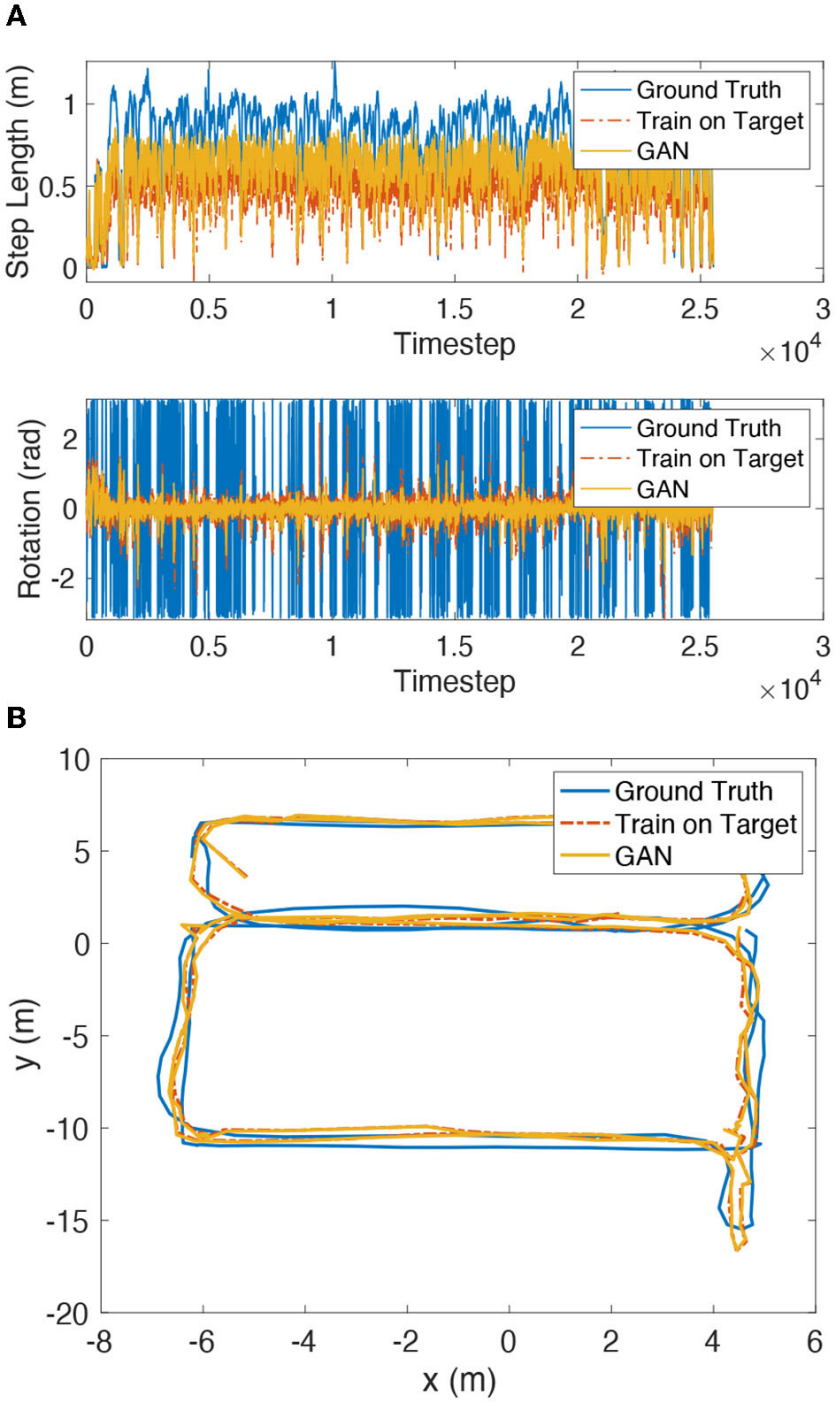
**(A)** Testset 1 transfer learning results of inertial encoder target. **(B)** Testset 1 transfer learning results of SmartFPS.

The test results of the inertial encoder network transfer learning algorithm are shown in Figure 10A and Table 4. The estimated step length and rotation angle of the inertial positioning network trained by the transfer learning method is closer to the ground truth values by 36.7–89.8% than those estimated by the network trained directly in the source domain. The test results of the wireless encoder transfer learning algorithm are shown in Table 4. Test set 2 and test set 3 showed significant improvement of over 38% on both encoder networks.

**Table 4:** Transfer learning results of inertial positioning network and wireless positioing network.

**Network**	**Test**	**Mean error**			
		**Train on source**	**Train on target**	**Transfer learning**	**Improvement (%)**
Inertial net [step length (m), rotation (rad)]	1	[0.334, 1.042]	[0.063, 0.903]	[0.207, 0.991]	[46.90, 36.69]
	2	[0.180, 0.891]	[0.048, 0.839]	[0.096, 0.866]	[63.86, 48.84]
	3	[0.101, 1.045]	[0.056, 1.001]	[0.079, 1.006]	[47.74, 89.80]
	4	[0.370, 0.893]	[0.089, 0.789]	[0.246, 0.843]	[44.37, 48.37]
Wireless net positioning error (m)	1	0.900	0.645	0.825	29.41%
	2	0.908	0.637	0.805	38.00%
	3	1.083	0.831	0.986	38.49%
	4	1.160	0.887	1.084	27.84%

The test results of SmartFPS are shown in Figure 10B. The update of the encoder weights of the fused localization network is performed concurrently with the update of the target domain dataset. The result shows that the localization trajectory before and after transfer learning is close to the ground truth trajectory, and the estimation of the model trained directly in the target domain.

The computation performance of transfer learning of each encoder is listed in Table 3. Due to the complexity of the cycle route and other routes, the average training time of transfer learning took up to over 2 s. However, the two transfer learning processes were done in the offline stage, which would occupy none of the resources during navigation.

## 6. Discussion

The choice of the number of beacons can directly influence the performance of the wireless encoder, as it can be seen as a continuous fingerprinting method. Since decreasing the number of beacons in some areas will remove signals with a high signal-to-noise ratio (SNR) in this area, the wireless encoder will fail or perform poorly. However, its influence on the whole fusion system is uncertain because of the assistance from the inertial sensor. To investigate how the number of beacons affects the performance of SmartFPS, we have considered two circumstances: (1) sparse deployment of beacons; (2) fewer or no beacons in one area.

**Figure 11:**
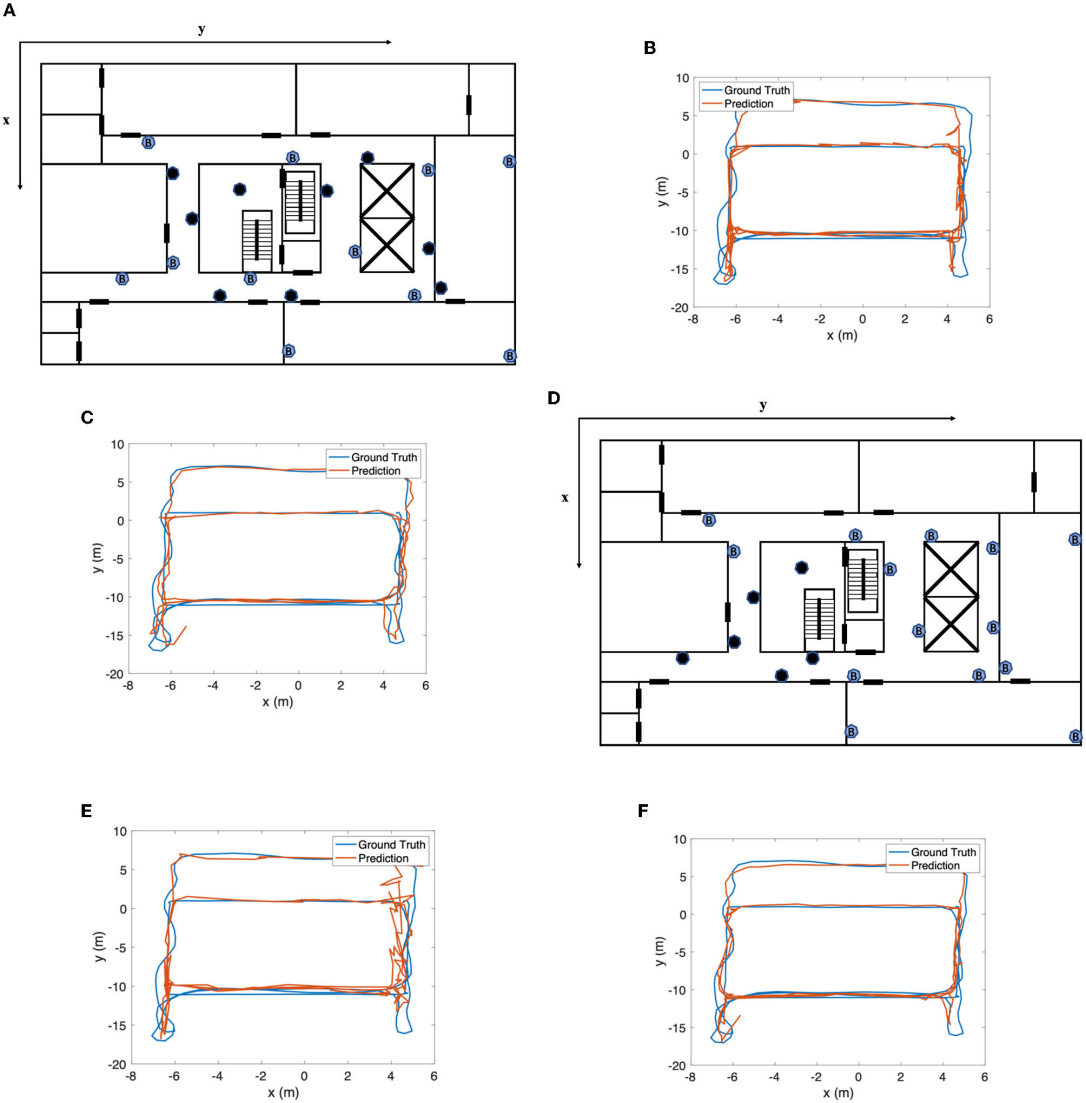
**(A)** Beacon layout for circumstance 1. **(B)** Wireless encoder result for circumstance 1. **(C)** SmartFPS result for circumstance 1. **(D)** Beacon layout for circumstance 2. **(E)** Wireless encoder result for circumstance 2. **(F)** SmartFPS result for circumstance 2.

For the first circumstance, since 20 beacons are deployed in our test field, we increase the interval between beacons by turning off several beacons evenly, as shown in Figure 11A, in which 9 of 20 beacons were turned off (colored in black) during the test. From the test result in Figures 11B, C, SmartFPS can still accurately track the pedestrian's location compared with the wireless encoder. The mean positioning errors for the wireless encoder and SmartFPS are 1.133 and 0.619 m, respectively. Compared with the result of fully functioning beacons, the accuracy of the wireless encoder was decreased by 59.8%. However, SmartFPS's performance was only downgraded by 18.3%.

For the second circumstance, we turned off six beacons deployed at one corner, as shown in Figure 11D. It can be seen from Figure 11E that the wireless encoder showed poor performance in this corner, with an overall positioning error of 1.362 m. SmartFPS was affected in the corner area as well, as shown in Figure 11F, but the overall performance is still much better than the wireless encoder. The mean positioning errors for the wireless encoder and SmartFPS are 1.271 and 0.776 m, respectively.

In general, decreasing the number of beacons has a much smaller influence on SmartFPS than the wireless encoder, indicating that the inertial encoder properly functioned in our fusion system.

## 7. Conclusion and future work

In this paper, an end-to-end deep learning-based wireless-inertial fusion positioning system, SmartFPS, and its transfer learning method are proposed. The features from sub-positioning networks are fused by a stateful LSTM network instead of filter-based methods. The multi-task learning method is adopted to simplify the training of SmarFPS and preserve the physical meaning of each sub-network. Furthermore, a GAN-based unsupervised transfer learning method is proposed to improve the performance of SmartFPS no matter the difference between pedestrians and devices. Field experiments are conducted, and the results show that our method significantly outperforms filter-based methods. SmartFPS showed high tolerance to larger noise factors compared with filter-based methods. Overall, our system can achieve an average positioning accuracy of 0.575 meters for different pedestrians and mobile phones.

For future work, specific techniques such as ZUPT and map fusion could be adapted to SmartFPS's architecture. Another interesting idea is replacing the inertial encoder with a variant-step inertial encoder, which can be derived from footstep detection techniques. When the foot lands on the ground, the inertial data sequence will generally show a large jitter. At present, machine learning algorithms have been studied in pedestrian footstep detection research, and the method based on the neural network has high estimation accuracy. Therefore, based on SmartFPS, the time series data can be segmented by footstep detection technology, and the inertial encoder can be replaced by a network structure without a fixed step length. Since the motion attitude of the pedestrian is relatively stable during each step, it can be estimated that this technique can further improve the positioning accuracy of SmartFPS.

## Data availability statement

The raw data supporting the conclusions of this article will be made available by the authors, without undue reservation.

## Author contributions

LH were responsible for providing the study idea, experiment design, experiment execution, result analyses, and manuscript draft. YZ and JY were responsible for supervision, review, and funding acquisition. All authors contributed to the article and approved the submitted version.
